# *catena*-Poly[[diaqua­zinc(II)]-μ-piper­azine-1,4-diacetato-κ^4^ *N* ^1^,*O* ^1^:*N* ^4^,*O* ^4^]

**DOI:** 10.1107/S1600536809045462

**Published:** 2009-11-11

**Authors:** Jian-Hong Bi

**Affiliations:** a Deparment of Chemistry and Chemical Engineering, Hefei Normal University, Hefei 230061, People’s Republic of China

## Abstract

The asymmetric unit of the title compound, [Zn(C_8_H_12_N_2_O_4_)(H_2_O)_2_]_*n*_, contains a Zn^II^ ion residing on an inversion center, half of a centrosymmetric piperazine-1,4-diacetate ligand (*L*) and a water mol­ecule. The Zn^II^ ion is *trans*-coordinated by two *N*,*O*-bidentate *L* ligands and by two water mol­ecules in a distorted octa­hedral geometry. In the crystal structure, inter­molecular O—H⋯O hydrogen bonds link polymeric chains into a three-dimensional supra­molecular structure.

## Related literature

For related structures, see: Wu & Mak (1996 ▶); Zhang & Chen (2003 ▶); Shen *et al.* (2006 ▶); Yang *et al.* (2008 ▶); Zhang *et al.* (2008 ▶).
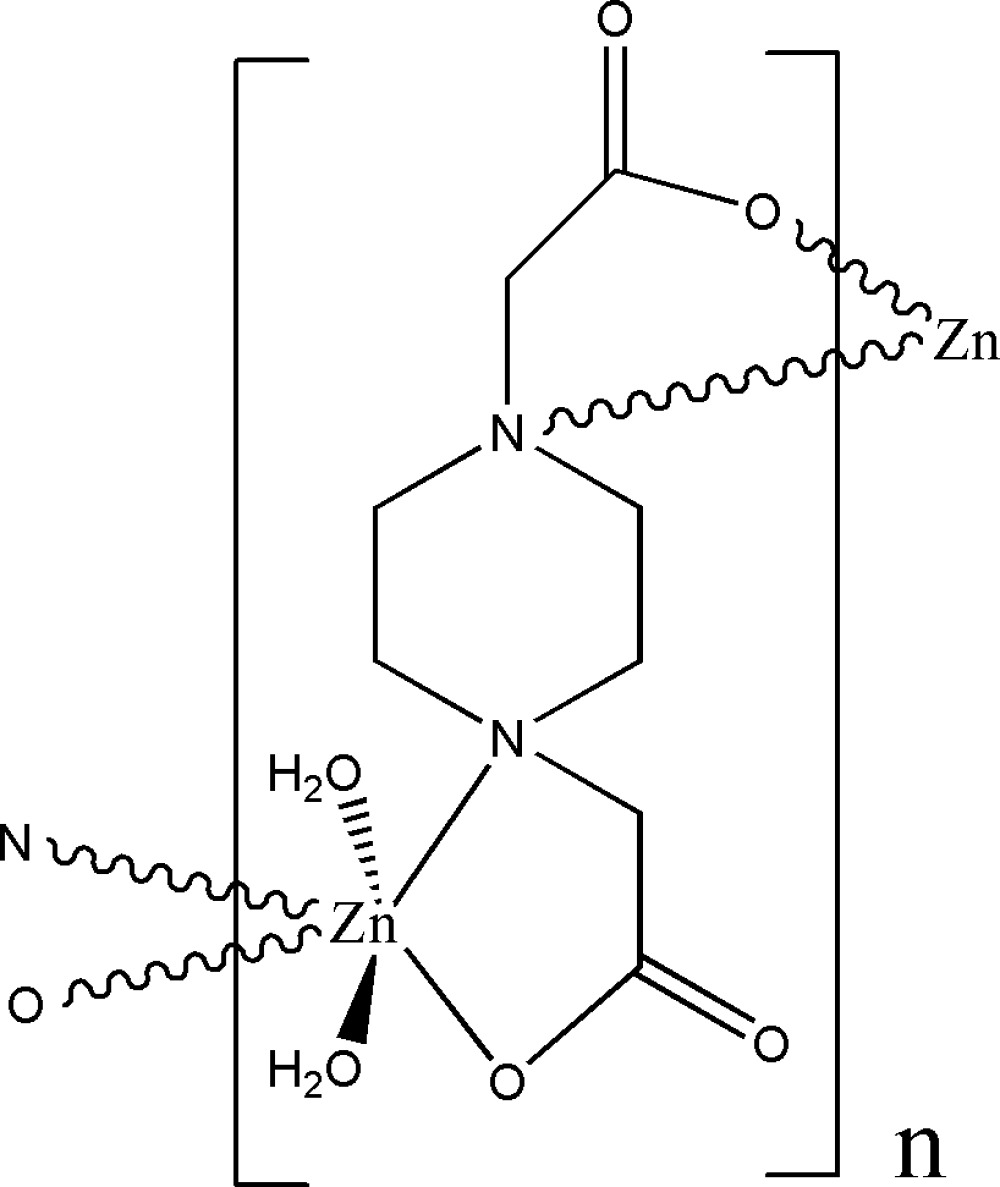


## Experimental

### 

#### Crystal data

[Zn(C_8_H_12_N_2_O_4_)(H_2_O)_2_]
*M* *_r_* = 301.62
Monoclinic, 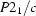

*a* = 6.3670 (1) Å
*b* = 7.3116 (10) Å
*c* = 11.9910 (1) Å
β = 101.438 (10)°
*V* = 547.13 (1) Å^3^
*Z* = 2
Mo *K*α radiation
μ = 2.27 mm^−1^
*T* = 291 K
0.30 × 0.15 × 0.12 mm

#### Data collection

Bruker SMART CCD area-detector diffractometer
Absorption correction: multi-scan (*SADABS*; Bruker, 2000 ▶) *T*_min_ = 0.517, *T*_max_ = 0.766
5254 measured reflections
1255 independent reflections
1173 reflections with *I* > 2σ(*I*)
*R*_int_ = 0.014

#### Refinement

*R*[*F* ^2^ > 2σ(*F* ^2^)] = 0.018
*wR*(*F* ^2^) = 0.050
*S* = 1.07
1255 reflections
87 parameters
2 restraints
H atoms treated by a mixture of independent and constrained refinement
Δρ_max_ = 0.35 e Å^−3^
Δρ_min_ = −0.17 e Å^−3^

### 

Data collection: *SMART* (Bruker, 2000 ▶); cell refinement: *SAINT* (Bruker, 2000 ▶); data reduction: *SAINT*; program(s) used to solve structure: *SHELXTL* (Sheldrick, 2008 ▶); program(s) used to refine structure: *SHELXTL*; molecular graphics: *SHELXTL*; software used to prepare material for publication: *SHELXTL*.

## Supplementary Material

Crystal structure: contains datablocks I, global. DOI: 10.1107/S1600536809045462/cv2645sup1.cif

Structure factors: contains datablocks I. DOI: 10.1107/S1600536809045462/cv2645Isup2.hkl

Additional supplementary materials:  crystallographic information; 3D view; checkCIF report

## Figures and Tables

**Table 1:** Hydrogen-bond geometry (Å, °)

*D*—H⋯*A*	*D*—H	H⋯*A*	*D*⋯*A*	*D*—H⋯*A*
O3—H3*A*⋯O1^i^	0.813 (19)	2.000 (19)	2.8060 (16)	172.9 (19)
O3—H3*B*⋯O1^ii^	0.826 (15)	1.927 (15)	2.7497 (15)	175 (2)

Symmetry codes: (i) 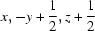; (ii) 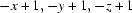.

**Fig. 1.**
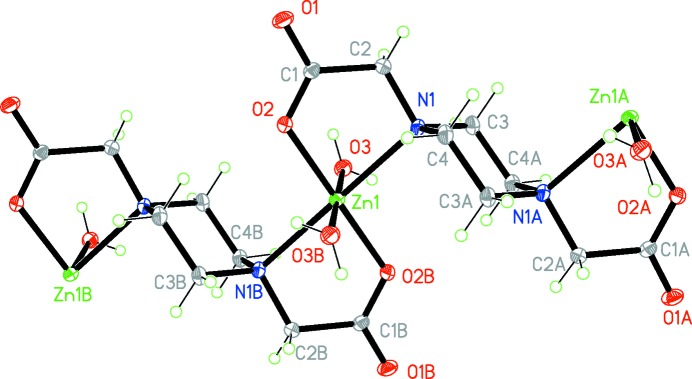
A portion of the polymeric chain in (I) showing 30% probability displacement ellipsoids and the atomic numbering [symmetry codes: (A) 1 - x, -y, 1 - z; (B) -x, -y, 1 - z].

## supplementary crystallographic information

### Comment

Researchers have shown their interest in design and synthesis of polydentate flexible ligands, which propagated a family of piperazine-based ligands, because the chair configuration of piperazine can reduce their coordination modes, which makes piperazine or its related species structurally or functionally directing polymeric constructions (Shen *et al.*, 2006; Wu *et al.*, 1996; Yang *et al.*, 2008; Zhang *et al.*, 2008; Zhang *et al.*, 2003). Herein, based on bridging 1,4-piperazinediacetic acid, we report the title compound and present its crystal structure.

The coordination geometry about Zn(II) center is shown in Fig.1. The Zn(II) center adopts an octahedral coordination geometry, in which two N atoms and two O atoms from two ligands are in the equatorial plane while the apical positions are occupied by two O atoms from water molecules. Intermolecular O—H···O hydrogen bonds (Table 1) link polymeric chains into three-dimensional supramolecular structure.

### Experimental

All solvents and chemicals were of analytical grade and were used without further purification. A mixture of H_2_*L*.2HCl (0.1 mmol), ZnCl_2_ (0.1 mmol), and water (10 ml) were heated in a 15-ml Teflon-lined vessel at 120 oC for 3 days, followed by slow cooling (5 oC h-1) to room temperature. After filtration and washing with H_2_O, colorless block crystals were collected and dried in air. Anal. Calcd.for C_8_H_16_N_2_O_6_Zn: C, 31.86; H, 5.35; N, 9.29. Found: C, 31.88; H,5.39; N, 9.22.

### Refinement

C-bound H atoms weregeometrically positioned (C—H 0.93–0.97 Å) and refined as riding, with U_iso_(H)=1.2U_eq_(C). Atoms H3A and H3B were located on a difference map, and refined with bond restraint O—H = 0.82 (2) Å as riding, with U_iso_(H)=U_eq_(O).

### Crystal data

**Table:**

[Zn(C_8_H_12_N_2_O_4_)(H_2_O)_2_]	*F*(000) = 312.0
*M**_r_* = 301.62	*D*_x_ = 1.831 Mg m^−^^3^
Monoclinic, *P*2_1_/*c*	Mo *K*α radiation, λ = 0.71073 Å
Hall symbol: -P 2ybc	Cell parameters from 1255 reflections
*a* = 6.3670 (1) Å	θ = 3.3–27.5°
*b* = 7.3116 (1) Å	µ = 2.27 mm^−^^1^
*c* = 11.9910 (1) Å	*T* = 291 K
β = 101.438 (1)°	Block, colourless
*V* = 547.13 (1) Å^3^	0.30 × 0.15 × 0.12 mm
*Z* = 2	

### Data collection

**Table:**

Bruker SMART CCD area-detector diffractometer	1255 independent reflections
Radiation source: fine-focus sealed tube	1173 reflections with *I* > 2σ(*I*)
graphite	*R*_int_ = 0.014
φ and ω scans	θ_max_ = 27.5°, θ_min_ = 3.3°
Absorption correction: multi-scan (*SADABS*; Bruker, 2000)	*h* = −8→8
*T*_min_ = 0.517, *T*_max_ = 0.766	*k* = −7→9
5254 measured reflections	*l* = −15→12

### Refinement

**Table:**

Refinement on *F*^2^	Primary atom site location: structure-invariant direct methods
Least-squares matrix: full	Secondary atom site location: difference Fourier map
*R*[*F*^2^ > 2σ(*F*^2^)] = 0.018	Hydrogen site location: inferred from neighbouring sites
*wR*(*F*^2^) = 0.050	H atoms treated by a mixture of independent and constrained refinement
*S* = 1.07	*w* = 1/[σ^2^(*F*_o_^2^) + (0.0264*P*)^2^ + 0.2196*P*] where *P* = (*F*_o_^2^ + 2*F*_c_^2^)/3
1255 reflections	(Δ/σ)_max_ < 0.001
87 parameters	Δρ_max_ = 0.35 e Å^−^^3^
2 restraints	Δρ_min_ = −0.17 e Å^−^^3^

### Special details

**Table:**

Experimental. The structure was solved by direct methods (Bruker, 2000) and successive difference Fourier syntheses.
Geometry. All e.s.d.'s (except the e.s.d. in the dihedral angle between two l.s. planes) are estimated using the full covariance matrix. The cell e.s.d.'s are taken into account individually in the estimation of e.s.d.'s in distances, angles and torsion angles; correlations between e.s.d.'s in cell parameters are only used when they are defined by crystal symmetry. An approximate (isotropic) treatment of cell e.s.d.'s is used for estimating e.s.d.'s involving l.s. planes.
Refinement. Refinement of *F*^2^ against ALL reflections. The weighted *R*-factor *wR* and goodness of fit *S* are based on *F*^2^, conventional *R*-factors *R* are based on *F*, with *F* set to zero for negative *F*^2^. The threshold expression of *F*^2^ > σ(*F*^2^) is used only for calculating *R*-factors(gt) *etc*. and is not relevant to the choice of reflections for refinement. *R*-factors based on *F*^2^ are statistically about twice as large as those based on *F*, and *R*- factors based on ALL data will be even larger.

### Fractional atomic coordinates and isotropic or equivalent isotropic displacement parameters (Å^2^)

**Table:**

	*x*	*y*	*z*	*U*_iso_*/*U*_eq_	
C1	0.3930 (2)	0.34321 (18)	0.39316 (11)	0.0202 (3)	
C2	0.2110 (2)	0.32565 (19)	0.45839 (13)	0.0246 (3)	
H2A	0.2484	0.3936	0.5290	0.029*	
H2B	0.0826	0.3804	0.4137	0.029*	
C3	0.0594 (2)	0.1341 (2)	0.58516 (12)	0.0223 (3)	
H3C	−0.0658	0.2127	0.5701	0.027*	
H3D	0.1581	0.1831	0.6505	0.027*	
C4	0.0075 (2)	0.0568 (2)	0.38757 (12)	0.0221 (3)	
H4A	0.0714	0.0528	0.3206	0.026*	
H4B	−0.1179	0.1350	0.3709	0.026*	
H3A	0.547 (3)	0.101 (3)	0.7111 (15)	0.043 (6)*	
H3B	0.607 (3)	0.255 (2)	0.6608 (17)	0.042 (6)*	
N1	0.16401 (17)	0.13433 (15)	0.48462 (10)	0.0198 (2)	
O1	0.3977 (2)	0.48174 (13)	0.33348 (10)	0.0299 (2)	
O2	0.53370 (15)	0.22018 (14)	0.40554 (9)	0.0263 (2)	
O3	0.59517 (17)	0.14245 (15)	0.65815 (9)	0.0265 (2)	
Zn1	0.5000	0.0000	0.5000	0.02027 (9)	

### Atomic displacement parameters (Å^2^)

**Table:**

	*U*^11^	*U*^22^	*U*^33^	*U*^12^	*U*^13^	*U*^23^
C1	0.0241 (6)	0.0172 (6)	0.0196 (6)	−0.0029 (5)	0.0052 (5)	−0.0009 (5)
C2	0.0228 (6)	0.0188 (7)	0.0346 (8)	0.0020 (5)	0.0116 (6)	0.0021 (6)
C3	0.0198 (6)	0.0264 (7)	0.0224 (7)	−0.0021 (5)	0.0080 (5)	−0.0027 (5)
C4	0.0188 (6)	0.0276 (7)	0.0205 (6)	−0.0001 (5)	0.0054 (5)	0.0020 (6)
N1	0.0185 (5)	0.0198 (5)	0.0228 (6)	−0.0020 (4)	0.0081 (4)	0.0006 (4)
O1	0.0418 (6)	0.0197 (5)	0.0307 (6)	0.0016 (4)	0.0131 (5)	0.0068 (4)
O2	0.0255 (5)	0.0224 (5)	0.0351 (6)	0.0040 (4)	0.0162 (4)	0.0083 (4)
O3	0.0328 (5)	0.0225 (5)	0.0270 (5)	−0.0011 (4)	0.0125 (4)	−0.0019 (4)
Zn1	0.02321 (13)	0.01652 (13)	0.02233 (13)	0.00056 (7)	0.00751 (9)	0.00295 (8)

### Geometric parameters (Å, °)

**Table:**

C1—O1	1.2439 (17)	C4—C3^i^	1.514 (2)
C1—O2	1.2575 (16)	C4—H4A	0.9700
C1—C2	1.5269 (18)	C4—H4B	0.9700
C2—N1	1.4773 (17)	N1—Zn1	2.3278 (11)
C2—H2A	0.9700	O2—Zn1	2.0042 (10)
C2—H2B	0.9700	O3—Zn1	2.1430 (11)
C3—N1	1.4884 (16)	O3—H3A	0.814 (15)
C3—C4^i^	1.514 (2)	O3—H3B	0.825 (16)
C3—H3C	0.9700	Zn1—O2^ii^	2.0042 (10)
C3—H3D	0.9700	Zn1—O3^ii^	2.1430 (11)
C4—N1	1.4864 (18)	Zn1—N1^ii^	2.3278 (11)
			
O1—C1—O2	123.53 (12)	C4—N1—C3	107.18 (10)
O1—C1—C2	118.08 (12)	C2—N1—Zn1	101.23 (7)
O2—C1—C2	118.34 (12)	C4—N1—Zn1	111.36 (8)
N1—C2—C1	113.26 (11)	C3—N1—Zn1	119.15 (8)
N1—C2—H2A	108.9	C1—O2—Zn1	119.19 (8)
C1—C2—H2A	108.9	Zn1—O3—H3A	115.2 (15)
N1—C2—H2B	108.9	Zn1—O3—H3B	121.7 (14)
C1—C2—H2B	108.9	H3A—O3—H3B	112 (2)
H2A—C2—H2B	107.7	O2^ii^—Zn1—O2	180.0
N1—C3—C4^i^	111.49 (11)	O2^ii^—Zn1—O3	86.18 (4)
N1—C3—H3C	109.3	O2—Zn1—O3	93.82 (4)
C4^i^—C3—H3C	109.3	O2^ii^—Zn1—O3^ii^	93.82 (4)
N1—C3—H3D	109.3	O2—Zn1—O3^ii^	86.18 (4)
C4^i^—C3—H3D	109.3	O3—Zn1—O3^ii^	180.0
H3C—C3—H3D	108.0	O2^ii^—Zn1—N1	100.60 (4)
N1—C4—C3^i^	110.87 (11)	O2—Zn1—N1	79.40 (4)
N1—C4—H4A	109.5	O3—Zn1—N1	87.66 (4)
C3^i^—C4—H4A	109.5	O3^ii^—Zn1—N1	92.34 (4)
N1—C4—H4B	109.5	O2^ii^—Zn1—N1^ii^	79.40 (4)
C3^i^—C4—H4B	109.5	O2—Zn1—N1^ii^	100.60 (4)
H4A—C4—H4B	108.1	O3—Zn1—N1^ii^	92.34 (4)
C2—N1—C4	109.09 (11)	O3^ii^—Zn1—N1^ii^	87.66 (4)
C2—N1—C3	108.39 (10)	N1—Zn1—N1^ii^	180.00 (6)

Symmetry codes: (i) −*x*, −*y*, −*z*+1; (ii) −*x*+1, −*y*, −*z*+1.

### Hydrogen-bond geometry (Å, °)

**Table:**

*D*—H···*A*	*D*—H	H···*A*	*D*···*A*	*D*—H···*A*
O3—H3A···O1^iii^	0.81 (2)	2.00 (2)	2.8060 (16)	173 (2)
O3—H3B···O1^iv^	0.83 (2)	1.93 (2)	2.7497 (15)	175 (2)

Symmetry codes: (iii) *x*, −*y*+1/2, *z*+1/2; (iv) −*x*+1, −*y*+1, −*z*+1.
